# Transition-Metal-Based Compounds for Electrochemical Energy Conversion Processes

**DOI:** 10.3390/ma16010067

**Published:** 2022-12-21

**Authors:** Andrzej Mikuła, Anna Kusior

**Affiliations:** Faculty of Materials Science and Ceramics, AGH University of Science and Technology, 30-059 Kraków, Poland

The era of ever-growing worldwide energy requirements demands the development of new methods of energy conversion, where the design of novel materials and the improvement of the efficiency of existing ones are of great importance. Transition-Metal-Based Compounds for Electrochemical Energy Conversion Processes is an open Special Issue of Materials, which welcomes original and novel research aimed at the most up-to-date research on this topic.

Transition-metal-based compounds, including intermetallic compositions, oxides, and chalcogenides, are characterized by several unique properties, mainly related to their crystal structure, electronic structure, and defect concentrations. The tendency of individual transition metals (TM) and the presence of arbitrarily occupied *d* orbitals allow the formation of a variety of polyhedrons differing by coordination number, which translates into a multitude of available crystallographic systems. On the other hand, TMs exhibit a high capacity for charge compensation and co-occurrence in multiple oxidation states, which affects the tunable electronic structure and vulnerability to defect formation in both cationic and anionic sublattices, respectively. The latter, in turn, makes it possible to direct transport properties (especially electrical ones) depending on the application. Lastly, TM-based compounds can be proceeded by means of a large variety of processes, allowing great potential for shape and surface engineering and tailorable morphologies, as well as various non-stoichiometric, multicomponent, and multiphase compositions.

The versatility of these compounds is manifested in their widespread use in energy-conversion-oriented technologies. One of the best examples is TM-based oxides belonging to the perovskite group, usually characterized by excellent thermomechanical, transport, and catalytic properties [1]. By skillfully assembling the chemical composition, it is possible to obtain extremely efficient materials, i.e., for fuel cell and photovoltaic technologies [2]. On the other hand, transition metal chalcogenides, offering a wide variety of available structures, susceptibility to defects, and high electroconductivity, have gained much interest for several applications, operating based on electrocatalytic processes [3,4].

In addition to the intrinsic properties of transition-metal-based compounds resulting directly from their structure, these materials are also capable of assembling into heterogeneous systems, allowing the optimization of synergistic effects and thus the modification of their final properties. An excellent example of this is the oxide–sulphide system, where the reciprocal arrangement of the valence and conduction bands causes an increase in the absorption band of the light prominence, as well as the tunneling of carriers and an increase in their lifetime [5]. As a result, an increase in the efficiency of cells operating on the basis of solar energy conversion processes is observed. Similar heterogeneous solutions can be found with various catalytical uses, where synergistic phenomena between different elements or compounds allow materials to be received with properties outside of the range typically observed for mixtures [4,6,7].

This Special Issue, entitled Transition-Metal-Based Compounds for Electrochemical Energy Conversion Processes, welcomes papers focused on transition-metal-based compounds for next-generation energy conversion devices, including sensors, photovoltaics, fuel cells, thermoelectrics, and catalysis. It is our pleasure to invite you to submit a manuscript for this Special Issue. Full papers, communications, and reviews are welcome.
